# Evaluation of Serum Cancer Antigen (CA)-125 Levels as a Biomarker for Ovarian Lesions: Correlation With Histopathological Diagnosis and Clinical Outcomes

**DOI:** 10.7759/cureus.65342

**Published:** 2024-07-25

**Authors:** Nanda J Patil, Avinash Mane, Atul B Hulwan, Mohammad Asim Khan, Huzaifa Umar

**Affiliations:** 1 Department of Pathology, Krishna institute of Medical Sciences, Krishna Vishwa Vidyapeeth “Deemed to be University”, Karad, IND; 2 Department of Pathology, Krishna institute of Medical Sciences, Krishna Vishwa Vidyapeeth "Deemed To Be University", Karad, IND; 3 Department of Pathology, Krishna Institute of Medical Sciences, Krishna Vishwa Vidyapeeth "Deemed To Be University", Karad, IND; 4 Department of Community Medicine, Mahatma Gandhi Medical College, Jaipur, IND; 5 Operational Research Center in Healthcare, Near East University, Nicosia, TUR

**Keywords:** clinical outcomes, histopathological diagnosis, biomarker, ca-125, ovarian cancer

## Abstract

Background: Ovarian cancer is a significant cause of morbidity and mortality among women worldwide. Early detection and accurate diagnosis are crucial for improving patient outcomes. Serum biomarkers, such as cancer antigen 125 (CA-125), have shown promise in aiding the diagnosis and monitoring of ovarian lesions.

Objective: This study aimed to assess the utility of serum CA-125 levels as a biomarker for ovarian lesions, correlating with histopathological diagnosis and clinical outcomes.

Methods: A prospective observational study was conducted, enrolling 144 female patients presenting with suspected ovarian lesions at a hospital or clinic. Demographic data, physical examination findings, imaging results, and serum CA-125 levels were collected at baseline. Patients underwent laparoscopic or surgical intervention for tissue biopsy or resection, and a histopathological examination was performed to confirm the diagnosis. Clinical outcomes, including response to treatment and disease recurrence, were monitored during follow-up visits.

Results: The baseline characteristics of the study population showed significant differences between participants with and without ovarian lesions. Older age (mean age 54.8 vs. 45.6) years; p < 0.001) and higher serum CA-125 levels (65.9 vs. 28.6 U/mL, p < 0.001) were associated with ovarian pathology. Histopathological analysis revealed benign cystadenoma as the most prevalent subtype (31.8%), followed by serous carcinoma (27.3%) and borderline tumors (22.7%). Clinical outcomes indicated favorable treatment responses in most patients, with 77.3% achieving complete remission and 15.9% experiencing recurrence. However, elevated CA-125 levels were significantly associated with poorer treatment response (p < 0.001) and higher rates of recurrence, suggesting its potential as a prognostic biomarker for ovarian lesions.

Conclusion: Serum CA-125 levels serve as a valuable biomarker for ovarian lesions, aiding in the diagnosis and monitoring of ovarian cancer. However, its utility is limited by its lack of specificity, particularly in differentiating between benign and malignant ovarian lesions. Integrating CA-125 with other clinical parameters and imaging modalities may enhance diagnostic accuracy and improve patient outcomes in ovarian cancer management.

## Introduction

Ovarian cancer represents a significant health challenge worldwide, ranking as the seventh most common cancer and the eighth leading cause of cancer-related deaths among women globally [1, 2]. According to GLOBOCAN 2020 statistics, 1.6% of new cases and 2.1% of deaths at all sites are attributable to ovarian cancer [1]. The disease is characterized by insidious onset and often presents at an advanced stage, resulting in a poor prognosis and limited treatment options [3]. Early detection and accurate diagnosis are essential for improving patient outcomes and reducing mortality rates associated with ovarian cancer [4]. In this context, serum biomarkers have emerged as valuable tools for the screening, diagnosis, and monitoring of ovarian lesions, offering noninvasive methods for detecting diseases and guiding treatment decisions [5].

Cancer antigen 125 (CA-125) is a biomarker that has garnered considerable attention in gynecological oncology. CA-125 is a glycoprotein antigen encoded by the MUC16 gene and is expressed in various epithelial tissues, including the ovarian epithelium [6]. Elevated serum CA-125 levels have been associated with various gynecological malignancies (fallopian tube cancer and endometrial cancer), particularly ovarian cancer, making it a clinically relevant biomarker for this disease [7]. However, CA-125 is not specific to ovarian cancer and can be elevated in benign conditions such as endometriosis, pelvic inflammatory disease, and uterine fibroids, as well as during menstruation and pregnancy [8]. Despite its limitations, CA-125 remains one of the most widely used biomarkers in clinical practice for ovarian cancer screening, monitoring treatment response, and detecting disease recurrence [9].

The utility of serum CA-125 level as a biomarker for ovarian lesions has been investigated in numerous studies, with conflicting findings regarding its diagnostic accuracy, sensitivity, and specificity [10]. Although elevated CA-125 levels have been associated with ovarian malignancies, including epithelial ovarian cancer, germ cell tumors, and sex cord-stromal tumors, the ability of biomarkers to differentiate between benign and malignant ovarian lesions remains suboptimal [11]. Furthermore, the use of CA-125 as a standalone diagnostic marker has limitations, particularly in premenopausal women and those with benign gynecological conditions, where false-positive results are common [11]. To minimize false-positive results in these cases, a higher cutoff, typically around 65 U/mL, may be recommended when using CA-125 as a diagnostic marker.

Despite these challenges, CA-125 continues to play a crucial role in the clinical management of ovarian cancer patients, guiding diagnostic workups, treatment selection based on the specific characteristics of the cancer, and the patient’s overall health and disease monitoring. Moreover, recent advances in biomarker research, including the development of novel biomarkers and multiplex assays, offer promising avenues for improving the accuracy and reliability of ovarian cancer diagnosis [7] [9]. Integrating serum CA-125 levels with other clinical parameters, imaging modalities, and molecular profiling techniques may enhance the diagnostic sensitivity and specificity of biomarker-based screening strategies, leading to earlier detection and improved outcomes in patients with ovarian cancer [12].

In this context, the study aimed to evaluate the utility of serum CA-125 levels as biomarkers for ovarian lesions, correlating them with histopathological diagnosis and clinical outcomes. By examining the association between CA-125 levels and ovarian lesion characteristics, including histological subtype, grade, and clinical stage, this study aimed to elucidate the diagnostic and prognostic implications of serum CA-125 levels in ovarian cancer management.

## Materials and methods

This study employed a prospective observational design to assess the utility of serum CA-125 levels as a biomarker for ovarian lesions among female patients. This study aimed to evaluate the association between serum CA-125 levels and ovarian lesion characteristics, as well as clinical outcomes. The study included female patients aged ≥ 18 years who presented with suspected ovarian lesions at the gynecology department. The inclusion criteria were clinical suspicion of ovarian lesions based on symptoms, physical examination findings, and imaging results. However, patients with a history of ovarian cancer or diagnosed ovarian lesions were excluded from the study to maintain consistency within the study population.

Inclusion criteria: female patients aged ≥ 18 years presenting at the gynecology department with suspected ovarian lesions were eligible for inclusion in this study. Suspected ovarian lesions were defined based on symptoms reported by the patients, physical examination findings, and imaging results such as pelvic ultrasound or magnetic resonance imaging (MRI). These symptoms may include pelvic pain, abdominal bloating, changes in urinary habits, or abnormal vaginal bleeding. Eligible participants were required to have the capacity to provide informed consent for study participation and to be willing to undergo serum CA-125 testing and subsequent evaluations.

Exclusion criteria: patients with a history of ovarian cancer or diagnosed ovarian lesions were excluded from the study to maintain homogeneity within the study population. Additionally, individuals who had been diagnosed with ovarian lesions prior to enrollment were not eligible for inclusion. This exclusion criterion aimed to ensure that the study focused on patients with suspected rather than confirmed ovarian lesions. Patients with known benign conditions (such as endometriosis or fibroids) that could independently elevate CA-125 levels were excluded from the study. Furthermore, patients who were unable or unwilling to provide informed consent for participation in the study were also excluded.

Convenience sampling was used to recruit participants from the gynecology department, ensuring the enrollment of eligible patients seeking evaluation for suspected ovarian lesions within the study setting. This sampling method facilitated efficient recruitment while ensuring that the study population was representative of the patient population seeking medical care for suspected ovarian lesions at hospitals or clinics. Before enrollment, eligible patients were provided with detailed information about the study objectives, procedures, and potential risks. Written informed consent was obtained from all the participants. Ethical approval was obtained from the institutional review board (IRB) before the commencement of the study. This study was conducted by the principles outlined in the Declaration of Helsinki. Patient confidentiality was strictly maintained, and all data were anonymized to protect participants’ privacy.

Comprehensive demographic information was gathered from each participant, including age, medical history, and relevant clinical details. This included details such as previous medical conditions, surgical history, family history of gynecological disorders, and current medications. Collecting these demographic data provided insights into the characteristics of the study population and helped identify potential confounding factors. A thorough physical examination, including a pelvic examination, was performed on each participant by qualified healthcare professionals. The pelvic examination aimed to assess the presence of ovarian lesions, evaluate their size, location, and characteristics, and identify any associated symptoms or signs, such as palpable masses or abdominal tenderness. This examination is crucial for initial clinical assessment and provides valuable information for subsequent diagnostic evaluations.

Patients underwent imaging studies, such as pelvic ultrasound or MRI, to visualize and characterize suspected ovarian lesions. Pelvic ultrasound is a commonly used imaging modality that allows for the visualization of ovarian structures and the detection of abnormalities such as cysts or tumors. MRI provides detailed images of pelvic structures and is particularly useful for assessing the extent and characteristics of ovarian lesions, including their size, morphology, and proximity to the adjacent structures. These imaging studies play a vital role in confirming the presence of ovarian lesions and guiding further diagnostic and therapeutic interventions.

Blood samples were collected from each participant to measure serum CA-125 levels using standard laboratory assays. CA-125 is a tumor marker that is frequently elevated in patients with ovarian cancer and other gynecological disorders. Measurement of serum CA-125 levels serves as a noninvasive diagnostic tool to assess the likelihood of ovarian lesions and monitor disease progression. This biomarker provided valuable information regarding the presence and severity of ovarian lesions and contributed to the overall diagnostic evaluation of study participants.

Patients diagnosed with suspected ovarian lesions underwent laparoscopic or surgical intervention for tissue biopsy or resection. Laparoscopic procedures involve the insertion of a thin, flexible tube with a camera (laparoscope) through a small incision in the abdomen to visualize and access the ovaries. Surgical interventions may include procedures such as ovarian cystectomy, salpingo-oophorectomy, or hysterectomy, depending on the nature and extent of the ovarian lesions. Tissue sampling through biopsy or resection allows the collection of ovarian tissue specimens for further histopathological evaluation. Ovarian tissue samples obtained from surgical or laparoscopic procedures were sent to the pathology department for histological examination and diagnosis. Experienced pathologists conducted a thorough microscopic evaluation of the tissue specimens to assess the cellular morphology, tissue architecture, and any pathological features indicative of ovarian lesions. Histopathological examination was performed to confirm the presence of ovarian lesions, classify them into specific histological subtypes (e.g., serous, mucinous, endometrioid), and determine the grade or level of malignancy if applicable. The histological diagnosis provided crucial information regarding the nature, extent, and severity of ovarian lesions, guiding subsequent treatment decisions and prognostic assessments. Additionally, histopathological examination allowed for the identification of concurrent or incidental pathologies within ovarian tissue samples. The histopathological evaluation findings were documented in detailed pathology reports, which formed the basis for clinical management and further patient follow-up.

Following the initial assessment and diagnosis, patients were subjected to regular follow-up evaluations in every 3 to 6 months for 2 years to monitor their clinical progress and outcomes. These follow-up assessments aimed to track the response to treatment, identify any recurrence of ovarian lesions, and evaluate the overall survival rates. Patients were scheduled for periodic visits to the clinic or hospital, typically at predetermined intervals based on the individual’s treatment plan and the nature of their ovarian lesions. During these follow-up visits, healthcare providers conducted comprehensive clinical examinations, including pelvic examinations and imaging studies, to assess the status of ovarian lesions and detect any signs of disease progression or recurrence. Any adverse events, treatment-related complications, or new symptoms were documented and managed accordingly. Regular monitoring of clinical outcomes allows healthcare professionals to tailor treatment strategies and provide appropriate interventions to optimize patient outcomes.

Serial measurements of serum CA-125 levels were performed during follow-up visits to monitor disease progression and treatment response. CA-125 is a tumor marker commonly used in the management of ovarian cancer and other gynecological disorders. Monitoring changes in serum CA-125 levels over time provides valuable insights into the efficacy of treatment interventions and the course of disease progression. For CA125, the normal upper limit was 35 U ml−1. Elevated CA-125 levels often indicate the presence or recurrence of ovarian lesions, whereas decreasing or stable CA-125 levels may suggest a favorable response to treatment or disease remission. These serial measurements of CA-125 levels were integrated into the overall clinical assessment of patients, complementing other diagnostic modalities and guiding clinical decision-making. Any significant changes in CA-125 levels were promptly investigated and appropriate interventions were implemented as necessary. Systematic monitoring of serum CA-125 levels during follow-up visits enabled healthcare providers to assess treatment efficacy, detect disease recurrence at an early stage, and optimize patient management strategies to improve clinical outcomes.

Descriptive statistics were used to summarize the demographic characteristics, serum CA-125 levels, histopathological findings, and clinical outcomes of the study population. Correlation analyses were conducted to assess the association between serum CA-125 levels and histopathological diagnosis. Survival analyses, such as Kaplan-Meier curves, were employed to evaluate the prognostic value of CA-125 levels in predicting clinical outcomes.

## Results

The baseline characteristics of the study population revealed notable differences between participants with and without ovarian lesions. First, the mean age of participants with ovarian lesions (54.8 years ± 7.2) was significantly higher than that of those without lesions (45.6 years ± 9.1), indicating that age may be a potential risk factor for the development of ovarian pathology (p < 0.001). Additionally, a higher proportion of postmenopausal women were observed in the ovarian lesion group (52.3%) than in the group without lesions (39.3%), suggesting that menopausal status could influence the likelihood of ovarian lesions (p = 0.012). Moreover, serum CA-125 levels were substantially elevated in participants with ovarian lesions (65.9 U/mL ± 22.7) compared to those without lesions (28.6 U/mL ± 12.4), indicating a strong association between elevated CA-125 levels and the presence of ovarian pathology (p < 0.001) (Table 1).

**Table 1:** Baseline Characteristics of the Study Population

Characteristic	Total (n=144)	Ovarian Lesions (n=88)	No Ovarian Lesions (n=56)
Age (years), mean ± SD	50.2 ± 8.6	54.8 ± 7.2	45.6 ± 9.1
Parity			
0	32 (22.2%)	18 (20.5%)	14 (25.0%)
1-2	58 (40.3%)	36 (40.9%)	22 (39.3%)
>2	54 (37.5%)	34 (38.6%)	20 (35.7%)
Menopausal status			
Premenopausal	76 (52.8%)	42 (47.7%)	34 (60.7%)
Postmenopausal	68 (47.2%)	46 (52.3%)	22 (39.3%)
CA-125 levels (U/mL), mean ± SD	45.7 ± 18.3	65.9 ± 22.7	28.6 ± 12.4
p-value	<0.001		

Histopathological diagnosis of ovarian lesions provides insight into the nature and composition of the identified lesions. Benign cystadenoma was the most prevalent histological subtype among participants with ovarian lesions, accounting for 31.8% of the cases. This finding suggests that benign cystic tumors are common among individuals with suspected ovarian pathologies. Additionally, serous carcinoma and borderline tumors were identified in 27.3% and 22.7% of the cases, respectively, indicating the presence of malignant and potentially precancerous lesions within the study population. This diverse histological spectrum highlights the heterogeneity of ovarian lesions and underscores the importance of accurate histopathological evaluation for appropriate diagnosis and management (Table 2).

**Table 2:** Histopathological Diagnosis of Ovarian Lesions

Histopathological Diagnosis	Frequency, n (%)	Mean CA-125 Level (U/mL) ± SD
Benign cystadenoma	28 (31.8%)	35.2 ± 12.1
Borderline tumor	20 (22.7%)	45.8 ± 15.3
Serous carcinoma	24 (27.3%)	78.5 ± 18.9
Mucinous carcinoma	16 (18.2%)	61.3 ± 14.7

Clinical outcomes of patients with ovarian lesions provide valuable insights into the prognosis and management of ovarian pathologies. A high proportion of patients with ovarian lesions demonstrated a favorable treatment response, with 77.3% achieving complete remission and 20.5% experiencing partial response. This finding suggests that most ovarian lesions are amenable to treatment interventions, resulting in favorable clinical outcomes. However, a subset of patients (2.2%) did not respond to treatment, indicating the presence of refractory or aggressive disease phenotypes that may require alternative therapeutic approaches. Furthermore, the recurrence rate of ovarian lesions was 15.9%, underscoring the importance of long-term surveillance and follow-up to monitor disease progression and detect recurrence at an early stage. The median overall survival of 48 months among patients with ovarian lesions highlights the potential for prolonged survival with appropriate management strategies (Table 3).

**Table 3:** Clinical Outcomes n: number of patients; %: percentage

Outcome	Ovarian Lesions (n=88)
Treatment response, n (%)	
Complete	68 (77.3%)
Partial	18 (20.5%)
No response	2 (2.2%)
Recurrence of lesions, n (%)	
Yes	14 (15.9%)
Overall survival (months), median (IQR)	48 (36-60)

The association between serum CA-125 levels and clinical outcomes provides valuable prognostic information regarding the utility of CA-125 as a biomarker of ovarian pathology. Higher CA-125 levels were significantly associated with poorer treatment response, as evidenced by the lower proportion of patients achieving complete remission and higher rates of lesion recurrence among individuals with elevated CA-125 levels (p < 0.001). This finding suggests that elevated CA-125 levels may indicate more aggressive disease phenotypes or resistance to conventional treatment. Additionally, the strong association between elevated CA-125 levels and lesion recurrence underscores the potential role of CA-125 as a predictive biomarker of disease progression and recurrence risk. Overall, these findings support the clinical utility of serum CA-125 levels in the risk stratification, treatment monitoring, and prognostic assessment of patients with ovarian lesions (Table 4).

**Table 4:** Association between CA-125 levels and clinical outcomes

Clinical Outcome	Serum CA-125 Levels (U/mL)	P-Value
Treatment response		
Complete response	60.2 ± 18.6	0.001
Partial response	68.4 ± 22.1	
No response	75.9 ± 25.3	
Recurrence of lesions		
Yes	78.5 ± 28.9	<0.001
No	55.6 ± 20.7	

## Discussion

Ovarian cancer is a significant cause of morbidity and mortality among women worldwide, with a late-stage diagnosis contributing to a poor prognosis and limited treatment options. Early detection plays a crucial role in minimizing the morbidity and mortality associated with cancer [1]. Therefore, there is an urgent need for genuine and reliable cancer indicators. PSA, CEA, and CA-125/MUC16 are frequently used cancer indicators, while exosomes, microRNA, and circulating tumor cells are emerging as a new source of biomarkers [13]. Biomarkers are integral in guiding treatment decisions by predicting disease outcomes and facilitating personalized treatment plans. They play a crucial role in monitoring disease progression, enabling adjustments to treatment strategies, and identifying early indicators of disease recurrence. This study aimed to evaluate the utility of serum CA-125 levels as biomarkers for ovarian lesions, correlating them with histopathological diagnosis and clinical outcomes. Serum CA-125 is a well-established tumor marker commonly used for the diagnosis and management of ovarian cancer [14]. Elevated CA-125 levels have been associated with various gynecological malignancies, including ovarian, endometrial, and fallopian tube cancers [7]. However, CA-125 is not specific to ovarian cancer and can be elevated in various benign conditions, such as endometriosis, pelvic inflammatory disease, and uterine fibroids. Despite its limitations, CA-125 remains a valuable tool in clinical practice for screening, monitoring treatment response, and detecting disease recurrence in ovarian cancer patients [15].

In this study, serum CA-125 levels were significantly elevated in participants with ovarian lesions compared with those without lesions, indicating a strong association between elevated CA-125 levels and ovarian pathology. Benign cystadenoma was the most prevalent histological subtype among participants with ovarian lesions, accounting for 31.8% of the cases. This finding suggests that benign cystic tumors are common among individuals with suspected ovarian pathologies. These findings are consistent with those of previous studies that demonstrated the utility of CA-125 as a biomarker for ovarian lesions. The observed correlation between serum CA-125 levels and histopathological diagnosis further supports the diagnostic value of CA-125 in identifying ovarian malignancies and guiding treatment decisions [7, 8, 16]. Histopathological evaluation remains the gold standard for diagnosing ovarian lesions and determining their malignant potential [17]. In this study, a diverse spectrum of ovarian lesions was identified, including benign cystadenomas, borderline tumors, and serous carcinomas. Benign cystadenomas were the most prevalent histological subtypes, followed by borderline and serous carcinomas. These findings highlight the heterogeneity of ovarian lesions and the importance of accurate histopathological diagnosis in guiding clinical management [18].

The correlation between serum CA-125 levels and histopathological diagnosis provides valuable insights into the biological behavior of ovarian lesions. Elevated CA-125 levels are significantly associated with malignant histological subtypes such as serous carcinomas, suggesting a potential role for CA-125 in predicting tumor aggressiveness and metastatic potential [19]. However, it is important to note that CA-125 levels can be influenced by various factors, including inflammation, menstruation, and other non-neoplastic conditions, which may limit its specificity as a standalone diagnostic marker [20].

The clinical outcomes of patients with ovarian lesions were evaluated to assess the prognostic value of serum CA-125 levels in predicting treatment response and overall survival. Most patients demonstrated a favorable treatment response, with a high proportion of patients achieving complete or partial remission. However, a subset of patients experience disease recurrence or demonstrate resistance to conventional treatment modalities, highlighting the challenges of managing advanced-stage ovarian cancer. Patients with elevated CA-125 levels are more likely to exhibit poorer treatment responses and higher rates of disease recurrence, suggesting a potential role for CA-125 as a prognostic marker for ovarian cancer [21]. These findings are consistent with those of previous studies demonstrating the prognostic significance of CA-125 in predicting clinical outcomes and survival in ovarian cancer patients [22]. Elevated CA-125 levels have been associated with advanced-stage disease, suboptimal debulking surgery, and an increased risk of recurrence, highlighting their utility as prognostic indicators of disease progression and survival [23].

Despite these promising findings, this study had several limitations. First, the study design may have introduced selection bias and confounding variables that could impact the interpretation of the results. The use of convenience sampling may introduce selection bias, as participants were recruited based on their availability and presentation at the gynecology department rather than through random selection from the population. This could affect the generalizability of the study findings to broader populations. Second, the sample size was relatively small, limiting the generalizability of the findings to larger populations. The age disparity between the case and control groups could potentially influence CA 125 levels, which might skew the study results. Additionally, the study focused exclusively on serum CA-125 levels and histopathological diagnosis, without considering other potential biomarkers or imaging modalities that could provide complementary diagnostic information. Future research directions should include prospective studies with larger sample sizes to validate the findings of this study and assess the long-term prognostic implications of serum CA-125 levels in patients with ovarian cancer. Furthermore, investigations into novel biomarkers, imaging techniques, and molecular profiling approaches may enhance the accuracy of ovarian cancer diagnosis, prognostication, and personalized treatment strategies.

## Conclusions

In conclusion, this study provides valuable insights into the utility of serum CA-125 levels as biomarkers for ovarian lesions, correlating them with histopathological diagnosis and clinical outcomes. Elevated CA-125 levels are significantly associated with ovarian pathology, malignant histological subtypes, and poor treatment response. These findings support the clinical utility of CA-125 in diagnosing ovarian cancer, monitoring treatment responses, and predicting clinical outcomes. However, further research is warranted to validate these findings and explore the potential implications of personalized treatment strategies and prognostic prediction models in ovarian cancer management.
